# Venesection in Opium Poisoning

**Published:** 1904-07-23

**Authors:** 


### VENESECTION IN OPIUM POISONING.

The proposal to bleed a patient suffering from severe poisoning by opium would in the present day seem to be a somewhat heroic one, and to carry it into operation in private practice argues considerable courage. Dr. Wm. A. Caskie[1] advocates this plan and is able to support his suggestion by a successful experience. The patient was a young woman who had taken more than an ounce of laudanum. When discovered, some hours after the administration of the poison, she was in a comatose condition and all attempts to rouse her proved unavailing. In these circumstances, the position seeming a hopeless one, it was determined to try the effect of bleeding, and this, as already stated, was followed by prompt improvement and subsequently by complete recovery. The suggestion is that the removal of blood means the removal of a certain proportion of the poison which is acting in a disastrous fashion on the nerve centres. Further, it must be remembered that the poison, though being in part eliminated by the kidney, is also excreted by the gastric and other mucous membranes. From these latter situations it may be, and frequently is, re-absorbed, thus gaining further opportunity to do mischief. Venesection, therefore, has a double value. It actually removes the poison, and it prevents any chance of re-absorption. There is certainly much more to be said for venesection in dangerous opium poisoning than for the practice of slapping, beating, and dragging about a semi-comatose and partially exhausted patient. These measures are often pushed to such an extreme that they do more harm than good.

[1] Brit. Med. Jour., March 19, 1904.

